# Conversion of Catalytically Inert 2D Bismuth Oxide Nanosheets for Effective Electrochemical Hydrogen Evolution Reaction Catalysis via Oxygen Vacancy Concentration Modulation

**DOI:** 10.1007/s40820-022-00832-6

**Published:** 2022-04-01

**Authors:** Ziyang Wu, Ting Liao, Sen Wang, Janith Adikaram Mudiyanselage, Aaron S. Micallef, Wei Li, Anthony P. O’Mullane, Jianping Yang, Wei Luo, Kostya Ostrikov, Yuantong Gu, Ziqi Sun

**Affiliations:** 1 School of Mechanical, Medical and Process Engineering, Queensland University of Technology, 2 George Street, Brisbane, QLD 4000 Australia; 2 Centre for Materials Science, Queensland University of Technology, 2 George Street, Brisbane, QLD 4000 Australia; 3 School of Earth and Atmospheric Sciences, Queensland University of Technology, 2 George Street, Brisbane, QLD 4000 Australia; 4 School of Chemistry and Physics, Queensland University of Technology, 2 George Street, Brisbane, QLD 4000 Australia; 5 Central Analytical Research Facility, Queensland University of Technology, 2 George Street, Brisbane, QLD 4000 Australia; 6 State Key Laboratory for Modification of Chemical Fibers and Polymer Materials, College of Materials Science and Engineering, Donghua University, Shanghai, 201620 People’s Republic of China

**Keywords:** Alkaline hydrogen evolution reaction, Bismuth oxide, Plasma irradiation, 2D materials, Oxygen vacancy

## Abstract

Oxygen vacancies (*V*_o_) in electrocatalysts are closely correlated with the hydrogen evolution reaction (HER) activity. The role of vacancy defects and the effect of their concentration, however, yet remains unclear. Herein, Bi_2_O_3_, an unfavorable electrocatalyst for the HER due to a less than ideal hydrogen adsorption Gibbs free energy (Δ*G*_H*_), is utilized as a perfect model to explore the function of *V*_o_ on HER performance. Through a facile plasma irradiation strategy, Bi_2_O_3_ nanosheets with different *V*_o_ concentrations are fabricated to evaluate the influence of defects on the HER process. Unexpectedly, while the generated oxygen vacancies contribute to the enhanced HER performance, higher *V*_o_ concentrations beyond a saturation value result in a significant drop in HER activity. By tunning the *V*_o_ concentration in the Bi_2_O_3_ nanosheets via adjusting the treatment time, the Bi_2_O_3_ catalyst with an optimized oxygen vacancy concentration and detectable charge carrier concentration of 1.52 × 10^24^ cm^−3^ demonstrates enhanced HER performance with an overpotential of 174.2 mV to reach 10 mA cm^−2^, a Tafel slope of 80 mV dec^−1^, and an exchange current density of 316 mA cm^−2^ in an alkaline solution, which approaches the top-tier activity among Bi-based HER electrocatalysts. Density-functional theory calculations confirm the preferred adsorption of H* onto Bi_2_O_3_ as a function of oxygen chemical potential (∆*μ*_O_) and oxygen partial potential (*P*_O2_) and reveal that high *V*_o_ concentrations result in excessive stability of adsorbed hydrogen and hence the inferior HER activity. This study reveals the oxygen vacancy concentration-HER catalytic activity relationship and provides insights into activating catalytically inert materials into highly efficient electrocatalysts.

**Supplementary Information:**

The online version contains supplementary material available at 10.1007/s40820-022-00832-6.

## Highlights

- Catalytically inert 2D Bi_2_O_3_ is activated for boosting electrochemical hydrogen evolution reaction (HER) via oxygen vacancy concentration modulation.
- The relationship between the varied oxygen vacancy concentrations and the corresponding HER activity is revealed by both experimental* V*_o_ verification and theoretical density-functional theory calculations.
- This work provides insights into activating catalytically inert materials into high-performance catalysts.

**Supplementary Information:**

The online version contains supplementary material available at 10.1007/s40820-022-00832-6.

## Graphical abstract

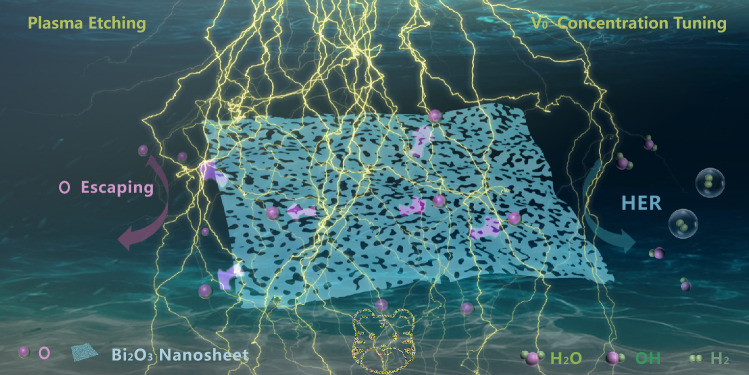

## Introduction

Green hydrogen has been identified as a promising energy source for sustainable development and an ideal candidate to replace traditional fossil fuels to address global environmental concerns [1]. Among various hydrogen production technologies, electrochemical water splitting encompassing the electrochemical hydrogen evolution reaction (HER) has been demonstrated to be a simple yet efficient route for the conversion of water into H_2_ [2–4]. Compared with HER in an acidic electrolyte, where the intermediate hydrogen is generated from proton reduction (H^+^  + e^−^ → H*), the alkaline-based reaction needs to overcome a higher energy barrier for the additional water dissociation step (H_2_O + e−  → H* + OH^−^) [5–7]. However, hydrogen production in alkaline media is desired for industrial production, due to the fact that often milder reaction conditions and higher anode catalyst activity are achievable for the catalysts towards the oxygen evolution reaction (OER) in alkaline electrolytes [8]. Noble metal electrocatalysts have so far attracted great attention for their high catalytic activity, but scarcity and their precious nature inhibit their large-scale application in electrocatalysis [9–12]. As a result, rational design and development of low-cost and high-performance electrocatalysts, e.g., transition metal oxide or carbon-based catalysts, for alkaline electrocatalysis, will play a vital role in the hydrogen economy [13–19].

Apart from exploring efficient earth-abundant metal oxide-based catalysts as potential Pt-free catalysts [20–22], various strategies have also been developed to enhance the activity of some existing electrocatalysts for alkaline conditions, such as multiple active sites tailoring [23, 24], electronic structure engineering [25–27], wettability control [28–30], etc., which can either optimize the interactions between the catalyst’s surface and the reaction intermediates or reduce the reaction energy barriers [31]. It should be noted that the oxygen or oxygen defective sites of metal oxide catalysts are strongly associated with electrocatalytic performance, especially in the water dissociation step of the HER. It has been well studied that the generation of oxygen vacancies (*V*_o_) can not only improve electronic conductivity but also change the interactions between the metal 3*d* and the O-2*p* band of the catalysts, which plays a vital role in the lattice oxygen involved in the OER [32]. Oxygen vacancies have also been reported as anchoring sites for constructing active single-atoms. In a recent work, Pt single atoms were anchored onto the *V*_o_ sites of MoO_2_ to achieve superior HER activity, where the *V*_o_ not only stabilizes the single atoms but also modulates the electronic structure of the catalysts [33]. Thus, creating *V*_o_ in metal oxides is an efficient strategy for modulating the surface electronic structure and tuning the intrinsic catalytic activity of electrocatalysts [34, 35]. Typical methods for creating *V*_o_ in the catalysts include thermal treatment, reduction reaction, doping, high-energy particle irradiation, etc. [36]. Although oxygen defects have been widely reported in enhancing the electrocatalysis performance, the role of oxygen vacancies in functionalizing the catalysts and the underlying mechanism are far from being fully understood. Thus, it is essential to create an effective model to accurately identify the role of *V*_o_ for the HER under alkaline conditions.

Bismuth (Bi), a *P*-block post-transition metal and a remarkably harmless element among the toxic heavy metals, has sparked interest in areas varying from medical to industrial chemistry [37]. Specifically, it has been demonstrated to be an outstanding electrocatalyst for carbon dioxide [38, 39] and nitrogen reduction [40–42], due to its inert HER activity and unfavorable free energy of hydrogen adsorption (∆*G*_H*_) which inhibits this competing reaction. Nørskov et al. have reported that the Bi catalyst has ultrahigh binding energy toward H* intermediates among various metal surfaces [43]. In addition, Bi_2_O_3_ is easily reduced into the metallic state and can accept electrons or loose oxygen ions to form a metal-like conductive surface [44, 45]. Compared with some other transition metals which usually need a high temperature reduction process or the use of a reducing agent [46], the property of Bi_2_O_3_ enables the formation of reductive defects under mild conditions. For this reason, defects can be conveniently introduced into bismuth oxide via facile methods [47]. Therefore, the inherently sluggish HER activity and the simplicity of constructing *V*_o_ of 2D Bi_2_O_3_ nanosheets endow this material as an ideal candidate to reveal the relationship between the concentration of *V*_o_ and HER performance.

Herein, based on 2D Bi_2_O_3_ nanosheets, we proposed a low-temperature plasma enabled approach to tune the oxygen vacancies concentration in the catalysts by plasma irradiation and investigated the corresponding alkaline HER performance to elucidate how the oxygen vacancies improve the alkaline activity of the catalyst, and tried to provide a principle to tune the inactive materials into active catalysts while maintaining their inherent chemical durability. This work discovered that the oxygen vacancy concentration is closely related with the alkaline HER catalytic activity of Bi_2_O_3_, but there is a saturation value of *V*_o_ to achieve the highest activity, over which the activity starts to drop. We then studied the relationship of the adsorption of the hydrogen intermediate onto the various active sites as a variation of partial oxygen pressure and the oxygen chemical potential by applying density functional theory (DFT) calculations. The theoretical investigation reveals that the single H* on O site (H*_O_) is the preferred adsorption model at a low *V*_o_ concentration whereas the adsorption of single H* on twin oxygen vacancies site (2*V*_o_ − H*_O_) is dominant at a high *V*_o_ concentration. However, the *V*_o_ concentration cannot be too high, otherwise the desorption energy of H* will be too high to desorb for the following step. As a result, the 2D Bi_2_O_3_ nanosheets with a medium *V*_o_ concentration and a detectable charge carrier concentration of 1.52 × 10^24^ cm^−3^ presented a superior HER performance with a small overpotential (174.2 mV at 10 mA cm^−2^) and a low Tafel slope (80 mV dec^−1^) but a high exchange current density of 320 mA cm^−2^ in 1 M KOH achieved in this HER inert catalyst.

## Experimental Section

### Materials

The chemicals used for the synthesis of 2D Bi_2_O_3_ nanosheets include bismuth nitrate pentahydrate (Bi(NO_3_)_3_·5H_2_O), polyethylene oxide-polypropylene oxide-polyethylene oxide (PEO_20_-PPO_70_-PEO_20_, Pluronic P123), ethylene glycol (C_2_H_6_O_2_, EG), Nafion solution (117 solution, Aldrich), and deionized (DI) water.

### Synthesis of Bi_2_O_2_CO_3_@Ni Foam (RT), Bi_2_O_3_@Ni Foam (Pl-0)

First, P123 (0.3 g) was dissolved in a mixed solution of ethanol (6 mL) and water (4.25 mL) (Solution A). Then, 13 mL EG was used to dissolve Bi(NO_3_)_3_·5H_2_O (0.2 g) to form homogeneous Solution B, which was mixed with solution A after 5 min ultrasonication. The mixed solution was kept at room temperature with an aging time of 24 h. Ni foam substrates were subsequently cleaned with 1 M HCl solution, acetone, ethanol, and DI water in an ultrasonic bath for 10 min. The cleaned Ni foam (1 × 1 cm^2^) was placed into the autoclave with the precursor solution, and the time of hydrothermal reaction maintained at 170 °C was set as 3 h for the uniform growth of 2D nanosheets. The sample was subsequently collected after complete cooling of the reactor, washed with ethanol twice and dried in an oven at 80 °C for 24 h (the as-synthesized sample was denoted as RT). The dried powders were then heat-treated in a furnace at 400 °C for 2 h in air with a ramping rate of 5 °C min^−1^ to get the bismuth oxide sample on Ni foam (Pl-0).

### Plasma Irradiation Treatment

The plasma treatment was conducted in an atmospheric-pressure quartz dielectric barrier discharge plasma reactor, where the quartz reaction chamber was sealed by a quartz lid cover. A CTP 2000 K power supply was employed to provide an amplitude of 26 kV and frequency of 9.1 kHz. A Rigol DS6104 oscilloscope was used to record the voltage and current changes. The feeding gas used in this study was N_2_. The Bi_2_O_3_ covered Ni foam samples at a size around 1 × 1 cm^2^ were placed at the center of the reactor and exposed with the desired time of 15, 30, and 60 min at a set power of 300 W. The samples after treatment were denoted as Pl-15, Pl-30, and Pl-60. Catalyst loadings after plasma treatment were weighted, which were about 4.8, 4.4, 4.4, 4.1, and 3.9 mg cm^−2^, respectively, for the samples of RT, Pl-0, Pl-15, Pl-30, and Pl-60. Andor SR-500 spectrometer was utilized to record the optical emission spectrum (OES) of the plasma. The Newton CCD camera with fiber optic cable were equipped on the device for recording and safety issue.

### Materials Characterizations

A field emission scanning electron microscope (FESEM, JSM-7001F, JEOL) was used to characterize the surface structure. A transmission electron microscope (TEM, JEOL 2100) was employed to characterize the morphology and crystal information. The sample surface element valence and chemistry property were measured by X-ray photoelectron spectroscopy (XPS, Kratos AXIS Supra photoelectron with Al-*Kα* radiation (hν = 1486.6 eV)). The calibration was performed by the containment carbon with a value of 284.8 eV. Grazing incidence diffraction (GID) geometry-based X-ray diffraction (XRD) was employed for the identifying of Ni foam loaded Bi_2_O_3_ nanosheets, and the spectrum was collected by a Rigaku Smartlab diffractometer (Cu radiation, 40 kV and 40 mA). The XRD patterns were collected at a scanning speed of 2° 2θ min^−1^ with the range from 5 to 90° 2θ maintained at a 0.02° step size. The phases of the samples were identified by JADE (V2010, Materials Data Inc.) and EVA (V5, Bruker) with PDF4 + 2021 reference database. Rietveld refinement was performed with a TOPAS package (V6, Bruker) for the quantitative analysis. Renishaw Raman microscope was used for the collecting of Raman spectra. The sample surface area was revealed by a Micromeritics Tristar II 3020 Surface Area and Porosity Analyzer. Brunauer–Emmett–Teller method was employed for the related surface area calculation. NT-MDT Solver Pro atomic force microscope (AFM) was employed to characterize the Bi_2_O_3_ nanosheets thickness before and after plasma treatment. Samples were prepared for electron paramagnetic resonance (EPR) spectroscopy by scratching Bi_2_O_3_ powder from the surface of the paramagnetic Ni foams. EPR spectrometer (Magnettech MiniScope MS400) with a benchtop setting was used to collect the EPR spectra. The X-band (*ν*) was 9.4 GHz, and the measurements were conducted at room temperature. For better comparation of the EPR results, the samples were tested with same weight (13 mg), filling almost 1.5 cm-height at the bottom of the sample tube (diameter = 3 mm).

### Electrochemical Measurements

A CHI760E workstation (CH Instruments, Inc.) was used to perform the electrochemical tests in a three-electrode-setup cell with a graphite counter electrode, a saturated calomel electrode (SCE) reference electrode, a 1 × 1 cm^2^ Bi_2_O_3_ loaded Ni foam working electrode. Conversion equation (*E*_RHE_ = *E*_SCE_ + 0.244 + 0.059 × pH) was used to calculate the potentials relative to reversible hydrogen electrode (RHE). Pt/C (20 wt%) at a mass load of 4 mg cm^−2^ was used as the benchmark to evaluate the HER activity. 1.0 M KOH solution was used as electrolyte, and the scan rate of the polarization measurement was 5 mV s^−1^. iR-correction (95%) was applied during electrocatalytic measurements. Electrochemical impedance spectroscopy (EIS) measurements were performed over 0.1 to 100,000 Hz at an overpotential of 10 mV. The Mott-Schottky measurements were performed between − 1 and 0.5 V (vs. SCE) at a frequency of 1000 Hz. Cyclic voltammetry curves (CVs) were collected within a non-Faradaic potential range under the open-circuit potential of the system for estimating the electrical double-layer capacitance (*C*_dl_) and the electrochemical surface area (ECSA).

### Theoretical Calculations

A Quantum-Espresso package with a spin-polarized DFT framework was employed for the theoretical calculations proposed in this work [48]. Ultrasoft pseudopotentials was used to describe the electron–ion interactions, and the generalized gradient approximation (GGA) with Perdew-Burke-Ernzerhof (PBE) functional was applied for the exchange-correlation interactions [49, 50]. Plane waves (PWs) basis sets were employed for the calculations of the Kohn–Sham (K–S) orbitals and the charge density. Bi_2_O_3_ (010) supercells in either defect free state or with one or two oxygen vacancies involved were built to study their hydrogen adsorption stability, where a vacuum space of 15 Å along the c direction of the supercell was designed to avoid the mirror interactions. The Brillouin zone with a Γ and a 5 × 5 × 1 *k*-point mesh was used for geometric optimization and electronic structure analysis [51]. 1 × 10^−7^ eV for the energy and 1 × 10^−4^ eV Å^−1^ for the force were set as the convergence criteria in structural optimization.

The stability of the hydrogen adsorption on either the defect free or the oxygen vacancies involving the Bi_2_O_3_ surface was calculated by using atomistic thermodynamics methods [52, 53]. The formation energies were defined as follows:

1 $$E_{f} = E\left( {n_{{{\text{Bi}}}} , n_{{\text{O}}} , n_{{\text{H}}} } \right) - n_{{{\text{Bi}}}} \mu_{{{\text{Bi}}}} - n_{{\text{O}}} \mu_{{\text{O}}} - n_{{\text{H}}} \mu_{{\text{H}}}$$

where the *E*(*n*_Bi_, *n*_O_, *n*_H_) denotes the total energy of the supercell containing the oxygen vacancies and adsorbed hydrogen, *n*_Bi_, *n*_O_, and *n*_H_ are the numbers of each species, and *μ*_*i*_ is the reference chemical potential of the species. To avoid the spontaneous formation of either the elemental solid or the gas, the chemical potential must be less than the total energy of their ground state, i.e., $\mu_{{{\text{Bi}}}} < E_{{{\text{Bi}}}}^{{{\text{Bulk}}}} {\text{and}} \mu_{{\text{O}}} < \frac{1}{2}E_{{{\text{O}}_{{2}} }}^{{{\text{Gas}}}}$. Meanwhile, the chemical potential of each constituent species must satisfy the relationship of:

2 $$2\mu_{{{\text{Bi}}}} + 3\mu_{{\text{o}}} < E_{{{\text{Bi}}_{{2}} {\text{O}}_{{3}} }}^{{{\text{Bulk}}}}$$

The chemical potential of oxygen was written as a thermodynamic expression of ideal gases depending on the pressure and temperature as follows:

3 $$\mu_{{\text{O}}} \left( {T,\rho } \right) = \mu_{{\text{O}}} \left( {T,\rho^{{0}} } \right) + 1/2kT\ln \left( {\rho_{{{\text{O}}_{{2}} }} /\rho_{{{\text{O}}_{{2}} }}^{0} } \right)$$

In which *ρ*^0^ is the reference state pressure. The chemical potentials *μ*_o_(*T*, *ρ*^0^) at the desired temperature and the reference pressure *ρ*^0^ were obtained from the thermochemical reference tables [54].

## Results and Discussion

### Characterization of Plasma Irradiated Catalysts

#### SEM and TEM Characterizations

**Fig. 1:**
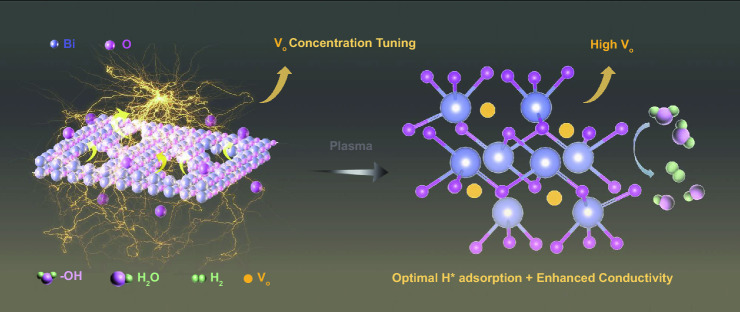
Schematic illustration of plasma irradiation on the formation of oxygen vacancies in 2D Bi_2_O_3_ nanosheets for electrocatalytic HER

The fabrication of 2D bismuth oxide nanosheets on Ni foam was carried out by a hydrothermal molecular self-assembly method [55], followed by high temperature calcination for conversion into Bi_2_O_3_ at 400 °C in air for 2 h (Fig. S1). Subsequently, plasma processing was conducted under N_2_ plasma with different irradiation durations (15, 30, and 60 min) to achieve different oxygen vacancy concentrations (Fig. 1). Gas-phase plasma usually interacts with the substrate surface via reactive intermediates derived from the gas. To characterize the activated species during the N_2_ plasma, an OES was recorded, and the typical band system at 300–400 nm (316, 337, 357, and 380 nm) evidently proved the second positive system from N_2_ during the plasma activation process (Fig. S2) [56]. Plasma has been reported to be an effective approach to create oxygen vacancies in metal oxides [57–59].

**Fig. 2:**
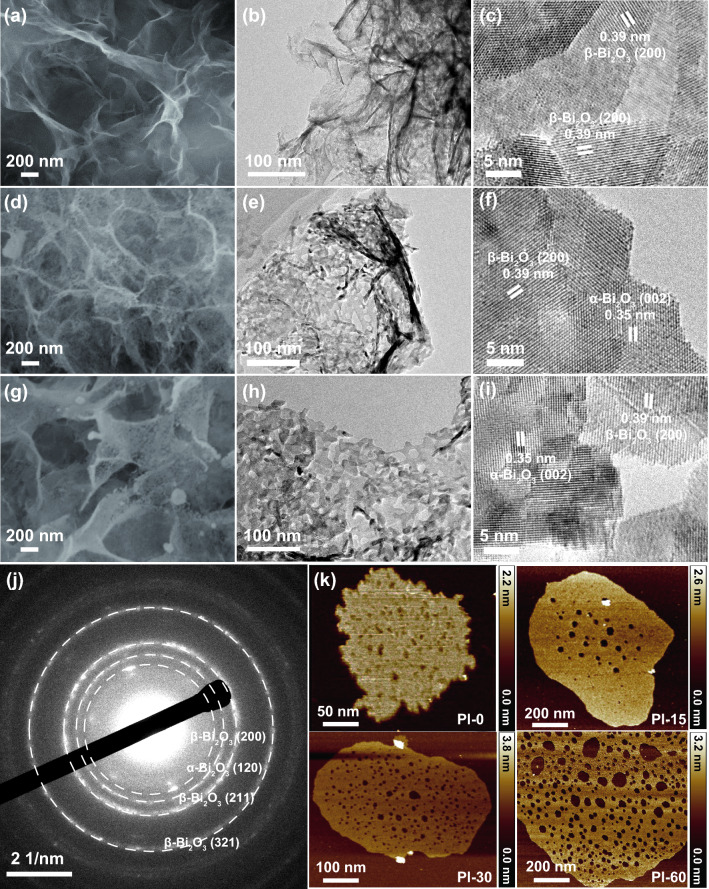
Morphology evolution of 2D Bi_2_O_3_ nanosheets upon plasma irradiations. SEM images (**a**, **d** and **g**), low-magnification TEM images (**b**, **e** and **h**) and high-resolution TEM images (**c**, **f** and **i**) of Bi_2_O_3_ before irradiation (Pl-0), irradiated for 15 min (Pl-15), and 30 min (Pl-30), respectively. SAED patterns (**j**), AFM images on the changes of thickness and pore sizes with different plasma irradiation durations (**k**)

To reveal the surface morphology evolution during the plasma treatment, SEM characterization was conducted. The as-synthesized ultrathin 2D Bi_2_O_3_ nanosheets on Ni foam demonstrated a uniform graphene-like structure (Fig. S3a, b), and no collapse occurred after calcination at 400 °C (Fig. 2a). The plasma irradiated Bi_2_O_3_ nanosheets had no significant change in their 2D form but the formation of through-holes with increasing numbers and pore size was observed with prolonged treatment time (Figs. 2 and S3c, d).

Low-magnification TEM images confirmed the structural changes with the plasma treatment (Fig. 2b, e, and h), where the 2D morphology of the samples were maintained with the formation of nanoholes. High-resolution TEM (HRTEM) detected phase transformation with the plasma treatment. For the as-synthesized Bi_2_O_3_ nanosheets without treatment (Pl-0, Fig. 2c), the lattice fringes with a spacing of 0.39 nm can be assigned to the (200) planes of *β*-Bi_2_O_3_ [45]. After plasma processing of 15 min (Pl-15, Fig. 2f) and 30 min (Pl-30, Fig. 2i), the (002) plane of the *β*-Bi_2_O_3_ phase with a spacing of 0.35 nm was identified. The co-existence of *α*-Bi_2_O_3_ and *β*-Bi_2_O_3_ phases implies that high-power plasma irradiation contributes to the atomic rearrangement of Bi_2_O_3_ [60]. The SAED patterns collected on the Pl-30 sample also confirmed the formation of the *α*-Bi_2_O_3_ phase (Fig. 2j), where the (120) planes for *α*-Bi_2_O_3_ were identified together with the lattice planes for *β*-Bi_2_O_3_ [61].

The thickness and the pore size of the 2D Bi_2_O_3_ nanosheets with plasma treatment at different durations were examined by AFM. As shown in Fig. 2k, increasing the irradiation time from 0 to 60 min resulted in the formation of more and larger nanopores in the nanosheets and a slight increase of the thickness of the nanosheets from 1.2 to 1.5 nm (Fig. S4a). The dark-field TEM observation with corresponding EDS mapping on Pl-30 indicate that the chemical distribution has not been disturbed by the plasma irradiation and uniform distributions of Bi, O, and C elements were clearly identified (Fig. S4b).

#### XRD and Raman Characterizations

**Fig. 3:**
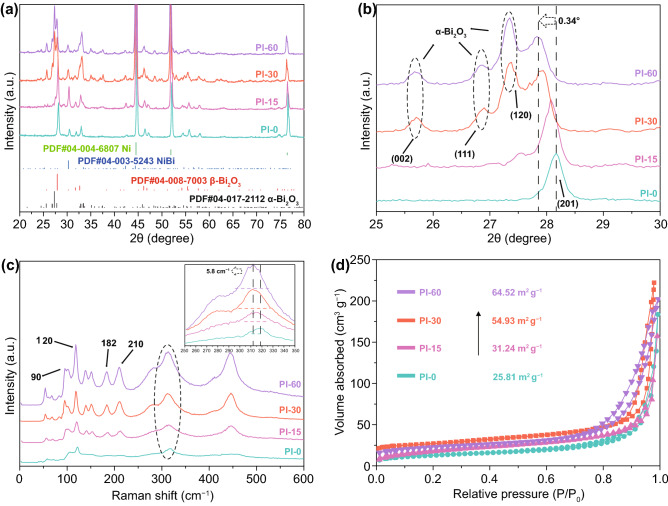
Phase evolution of 2D Bi_2_O_3_ nanosheets upon plasma irradiations. XRD spectrum (**a**), locally enlarged image of XRD patterns (**b**), Raman spectrum with the inset showing the peak shifting and broadening (**c**), and N_2_ adsorption and desorption spectrum (**d**) of samples Pl-0, Pl-15, Pl-30, and Pl-60

To further evaluate the phase transformation during the plasma irradiation process, XRD data were collected for qualitative and semi-quantitative analysis of the phases. As shown in Fig. 3a, in the Pl-0 sample, the *β*-Bi_2_O_3_ (PDF#04-008-7003, P-421c, *a* = 0.7755 nm, *c* = 0.5659 nm) phase dominated in the Bi_2_O_3_ nanosheets co-existing with traces of NiBi (PDF#04-007-2591) and the Ni metal substrate (PDF#04-004-6807). After 15 min of plasma treatment, only a very small amount of the α-Bi_2_O_3_ phase was detected (PDF#04-017-2112, P21/c, *a* = 0.5854 nm, *b* = 0.8165 nm, *c* = 0.7508 nm, *β* = 112.82°) (Fig. S5). However, when the treatment time increased to 30 min and 60 min, the ratios of *α*-Bi_2_O_3_ to *β*-Bi_2_O_3_ were 1.00 and 1.32 (Fig. S5), respectively, which could be the result of the continuous escape and rearrangement of O atoms during the plasma treatment [62]. It is interesting to note that, with a substantial increase of plasma etching time, an obvious shift of the (201) plane of *β*-Bi_2_O_3_ and the appearance of new peaks for the (002), (111), and (120) planes of *α*-Bi_2_O_3_ were identified, which were the result of phase transformation and lattice distortion (Fig. 3b). This result implies the expansion of the lattice along specific planes, due to oxygen vacancies generation activated by the plasma process [63]. Based on the TEM characterizations, the nanosheets presented a polycrystalline feature. The average crystalline size obtained from the XRD patterns by Rietveld refinement are of 41.2 and 25.7 nm for *α*-Bi_2_O_3_ and *β*-Bi_2_O_3_, respectively, which are coincided with the TEM observations.

Raman characterization was also conducted to identify the surface changes introduced by the plasma (Fig. 3c). For sample Pl-0, the Bi–O stretching modes at 90 and 120 cm^−1^ from *β*-Bi_2_O_3_ were clearly observed. It should be noted that the vibrational bands at 90, 120, and 316 cm^−1^ are shared by both *β*-Bi_2_O_3_ and *α*-Bi_2_O_3_, while the newly formed peaks at 182 and 210 cm^−1^ were as a result of the phase transformation into the monoclinic phase *α*-Bi_2_O_3_ [64]. Compared with Pl-0, the plasma processed samples demonstrated a red shift and peak broadening, as shown in the inset in Fig. 3c, which are usually associated with structural softening induced by heating, defects, etc., in the crystal structure [65, 66]. Therefore, both XRD and Raman characterizations confirmed the formation of oxygen vacancies in the Bi_2_O_3_ lattice. Owing to the creation of a large number of pores in the nanosheets, the specific surface area increased stepwise from 25.8 to 64.5 m^2^ g^−1^ upon prolonging the treatment time to 60 min (Fig. 3d).

#### XPS, EPR and Mott-Schottky Measurements on Oxygen Vacancy Concentrations

**Fig. 4:**
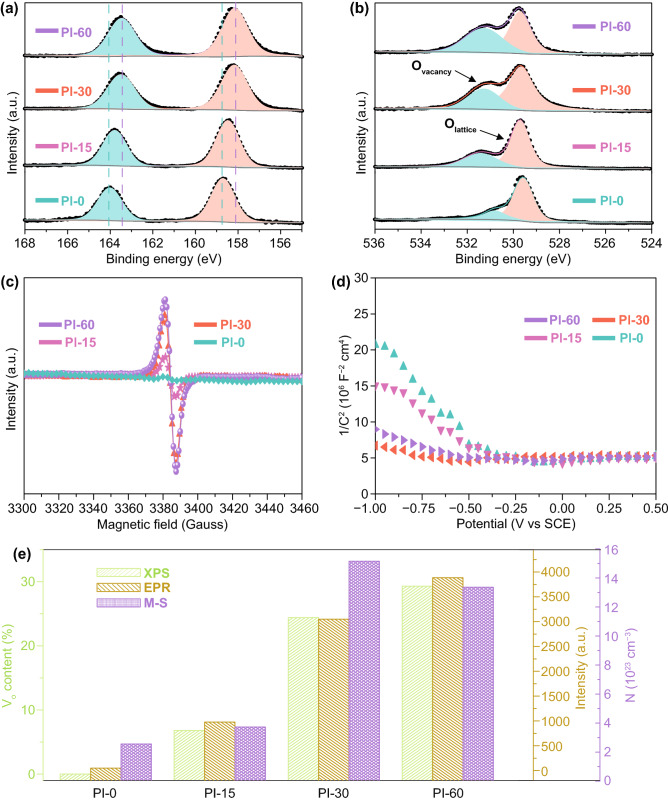
Chemical states and oxygen vacancy evolution of 2D Bi_2_O_3_ nanosheets upon plasma irradiations. High resolution XPS spectra of Bi 4*f* (**a**) and O 1s (**b**) bands, electron paramagnetic resonance (EPR) spectra (**c**), the Mott-Schottky plots (**d**) of Pl-0, Pl-15, Pl-30, and Pl-60 samples. The variation of oxygen vacancy concentration of 2D Bi_2_O_3_ with plasma irradiation time calculated based on the XPS, EPR, and Mott-Schottky measurements (**e**)

The chemical compositions and the defects of the 2D Bi_2_O_3_ nanosheets after plasma processing were identified by XPS measurements. The element survey spectrum indicated the presence of Ni, Bi, and O on the surface (Fig. S6). Even though N_2_-plasma has been used in this treatment, no N-doping was detected in all treated samples. In the Bi 4*f* spectrum (Fig. 4a), two peaks at 158.7 and 164 eV (∆*E* = 5.3 eV) assigned to the spin-orbit doublet of Bi 4*f*_7/2_ and Bi 4*f*_5/2_ for Bi^3+^ were observed on Pl-0. Upon plasma treatment, these two peaks shifted to lower binding energy. For the Pl-60, the peaks were located at 158.2 and 163.5 eV, corresponding to a 0.5 eV shift. This type of down shifting is due to lowering the coordination of Bi atoms by the formation of plasma induced oxygen vacancies [58, 67].

The O 1s core levels can provide more information on the formation of oxygen vacancies (Fig. 4b), where the O 1s state located at around 530.8 eV is associated with *V*_o_ or more accurately the change of chemical states of the lattice oxygen resulted by the formation of oxygen vacancies, and the one at 529.6 eV corresponds to lattice oxygen [62, 68]. Although the assignment of *V*_o_ has been disturbed by attached the hydroxyls, water molecules, and organic contaminants [69], the same fabrication, storage, and treatment conditions of the samples ensure that the increase of the high-energy core level of O 1s after plasma irradiation were ascribed by surface oxygen vacancies formation [58, 70, 71]. Therefore, we did not deconvolute the oxygen peak at 530–533 eV but exclude the contributions originated from the hydroxyls and the adsorbed water molecules by taking the non-treated sample Pl-0 as the baseline to calculate the generated oxygen vacancy concentrations resulted by the plasma irradiations. It is clear that the intensity and the proportion of the deconvolution associated with *V*_o_ increased significantly with the prolonged plasma treatment, corresponding to the increase of oxygen vacancy concentrations to 29.3% after exposing under plasma up to 60 min.

X-band EPR spectroscopy was also utilized to assess the formation of oxygen vacancies (Fig. 4c) [72]. The characteristic signal observed at g ≈ 2.001 is consistent with the formation of *V*_o_ defects upon plasma etching. The observed increase of the EPR signal intensity with plasma treatment time suggested that the number of oxygen vacancies is proportional to the treatment time. Table S2 displays the detailed values of the EPR variations.

The Mott-Schottky (M-S) test can provide quantitative information about the effective charge carriers generated within the semiconductors, and the increase of the carriers generated by the plasma irradiation could be considered as the generation of oxygen vacancies. Figure 4d presents the carrier density (*N*) as a function of the applied bias voltages recorded in the M-S tests, and *N* can be evaluated from the slope of M-S curves based on Eq. (4) [62].

4 $$N = \frac{2}{{e\varepsilon \varepsilon_{0} }} \left( {\frac{{{\text{d}}C^{ - 2} }}{{{\text{d}}V}}} \right)^{ - 1}$$

Here, *ε* is the dielectric constant of the material, which is 18.2 for Bi_2_O_3_ [73, 74]; *ε*_0_ is the vacuum permittivity (8.85 × 10^−12^ F m^−1^); *e* is the electron charge (1.6 × 10^−19^ C); and *V* is the applied bias. Figure 4d shows the plot of 1/*C*^2^ vs *V* of different samples measured in 1 M KOH electrolyte. The negative slopes indicate the *p*-type semiconductor behavior of Bi_2_O_3_ [75]. The calculated charge carrier density *N* for the 30 min treated Bi_2_O_3_ (Pl-30) is 1.52 × 10^24^ cm^−3^, which is nearly 1 order of magnitude higher than that of Pl-0 (2.53 × 10^23^ cm^−3^), confirming the significant increase of *V*_o_ upon prolonging the plasma treatment. A further increase in irradiation time to 60 min, resulted in the carrier density decreasing slightly to 1.34 × 10^24^ cm^−3^, although a higher *V*_o_ concentration was indicated in both the XPS and EPR characterizations in Pl-60, which may be a result from over irradiation induced phase transformation and structure deformation, which may hinder electron transfer as observed previously in Xiao’s work [76]. Figure 4e summarizes the relationship between the plasma induced *V*_o_ concentration and the irradiation time evaluated by these different approaches. Clearly, the *V*_o_ concentration calculated from XPS and EPR confirmed a nearly linear relationship with plasma irradiation time, demonstrating the effectiveness of the plasma treatment on generating *V*_o_ in Bi_2_O_3_ nanosheets.

### Electrocatalysis Performance of the Catalysts

**Fig. 5:**
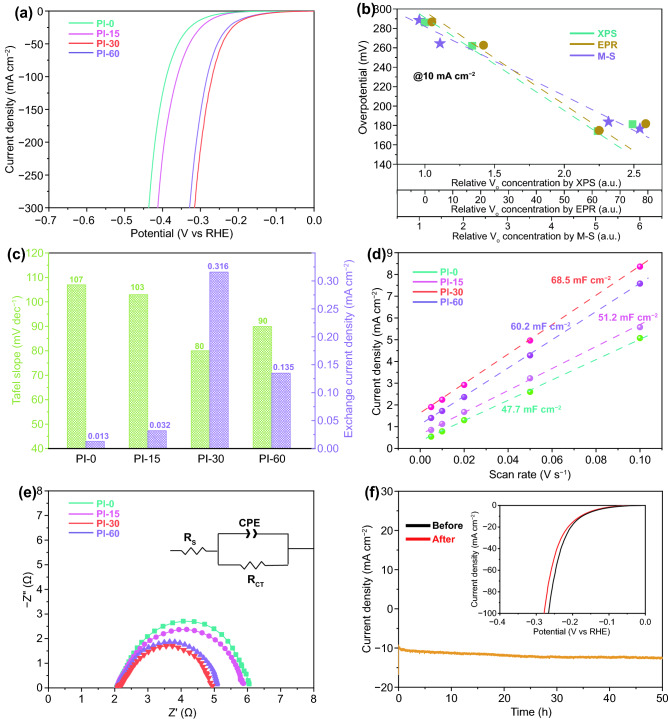
Electrocatalysis performance of 2D Bi_2_O_3_ nanosheets in 1.0 M KOH. Polarization curves collected at a scan rate of 5 mV s^−1^ (**a**). The relationship of the overpotentials needed to reach *j* = 10 mA cm^−2^ and the relative oxygen vacancy concentrations obtained from EPR, XPS and EPR measurements (**b**). Tafel slopes and exchange current densities for 2D Bi_2_O_3_ nanosheets with different oxygen vacancy concentrations (**c**). Calculated *C*_dl_ plots of 2D Bi_2_O_3_ nanosheets at 10 mA cm^−2^ (**d**). EIS measurements on different 2D Bi_2_O_3_ nanosheets. (**f**) Stability test on the Pl-30 up to 50 h (**e**)

The effect of tailored *V*_o_ on the electrocatalytic performance of the HER-inert Bi_2_O_3_ was evaluated in a 1 M KOH electrolyte with a standard three electrodes configuration. Figure 5a exhibits the linear sweep voltammetry (LSV) curves of the samples with the different plasma irradiation time, namely, with the different *V*_o_ contents. It is interesting that the Bi_2_O_3_ treated for 15–30 min presented significantly improved HER performance, and particularly, the Pl-30 sample showed the lowest onset potential after iR-compensation, demonstrating its lowest energy barrier to trigger the HER. To reach a current density of 10 mA cm^−2^, the requested overpotentials were 286.1, 262.1, 174.2, and 181.2 mV, respectively, for Pl-0, Pl-15, Pl-30, and Pl-60, respectively. The Pl-30 sample exhibited the lowest overpotential to reach 10 mA cm^−2^, which is more than a 110 mV improvement compared with the as-synthesized catalyst. While the HER activity of Bi_2_O_3_ after plasma treatment is still lower than that of a commercial Pt/C electrocatalyst (Fig. S7), the tuned Bi_2_O_3_ material exhibited promising potential for high current density catalysis, and only an overpotential of 310 mV is required to reach a current density of 300 mA cm^−2^. Most critically, this investigation reveals the success of the strategy of tuning the HER-inert catalysts into active electrocatalysts via oxygen vacancy modulation. Compared with previously very few reported Bi-based electrocatalysts for alkaline HER (Fig. S8), the plasma induced *V*_o_ activated 2D Bi_2_O_3_ presented the best performance.

For a better understanding on how *V*_o_ concentration contributes to the HER activity, we plotted the overpotential at 10 mA cm^−2^ as a variation of the *V*_o_ concentration measured by using XPS, EPR, and M-S techniques (Fig. 5b). A near linear relationship between the overpotential and the *V*_o_ in the Pl-0, Pl-25, and Pl-30 catalysts was identified for the data acquired by these methods, and Pl-30 presented the best performance. However, the Pl-60 which possess the highest absolute *V*_o_ identified by XPS and EPR exhibited decreased activity toward the HER. From this result, we can understand that there is a saturation level for the *V*_o_ generated in these materials [77]. If we look at the charge carrier density measured by the M-S test, a very close linear relationship exists between the overpotential and the charge carrier density for all samples, which reveals that only the effective carrier that can move freely can contribute to the HER catalysis, and therefore, the M-S measurement could be a more reliable method to identify the available active *V*_o_ sites for electrocatalysis. Therefore, Pl-60 possessed the highest *V*_o_ concentration, but some *V*_o_ are trapped and combined to become unmovable “dead” defects which cannot contribute to the HER.

The HER kinetics as a function of *V*_o_ concentration can also be determined from the Tafel slopes and the exchange current density (Figs. 5c and S9). The Pl-30 catalyst presented the lowest Tafel slope of 80 mV dec^−1^, which is comparable to Pl-60 (90 mV dec^−1^) but much smaller than that of Pl-0 (107 mV dec^−1^) and Pl-15 (103 mV dec^−1^), and the highest exchange current density (316 mA cm^−2^), a descriptor of catalytic activity, among all the samples. The Tafel slope can be used to address the elementary steps and the rate determining steps during the catalytic reactions [78]. For the HER electrocatalysis in alkaline condition involving both a water dissociation step and the adsorption and desorption process of hydrogen intermediates, water dissociation could be the rate determining step when its value is around 120 mV dec^−1^ [78, 79]. Here, the relatively lower Tafel slope of 80 mV dec^−1^ suggests that the water dissociation step became more favorable in the *V*_o_ concentration tuned electrocatalysts. As observed in the LSV measurements, a proper *V*_o_ concentration is critical for alkaline HER. Pl-30 with the most suitable *V*_o_ concentration presented the most favorable HER kinetics and the highest catalytic activity to deliver the most optimal HER reactivity.

The electrochemically active surface area (ECSA) provides a connection between the coverage of active sites and the catalytic activity, which can be estimated from the double-layer capacitance (*C*_dl_) through CVs measured within a non-Faradaic region at different scan rates. Figure 5d displays the *C*_dl_ calculated based on a series of CV measurements on the 2D Bi_2_O_3_ catalysts (Fig. S10), in which a higher *C*_dl_ means the higher available electrochemically active surfaces during HER due to the positive correlation between the *C*_dl_ and the ECSA. In this case, the Pl-30 showed the highest *C*_dl_ value of 68.5 mF cm^−2^, which is superior to Pl-60 (60.2 mF cm^−2^), Pl-15 (51.2 mF cm^−2^), and PL-0 (47.7 mF cm^−2^). This result indicates that the plasma processing can increase the number of electrochemically active sites, but excessive irradiation decreases the effectiveness of the catalysts even with an increased physical surface area. In addition, the superhydrophilic surfaces of the samples (Fig. S11) associated with the formation of oxygen vacancies also contributed to more active reaction sites during the HER process.

As an important indicator for the electrode kinetics of the HER, EIS provides detailed information on charge transfer kinetics. Figure 5e shows the Nyquist plot with an equivalent circuit and the simulated ohmic resistance (*R*_s_), charge transfer resistance (*R*_ct_) as well as the constant phase element (*R*_CPE_) to illustrate the reaction rates. It can be deduced from the *R*_ct_ values of Pl-0 (4.1 Ω), Pl-15 (3.9 Ω), Pl-30 (2.9 Ω), and Pl-60 (3.0 Ω) that the 30 min plasma irradiated sample displayed the highest charge transfer kinetics as a result of the plasma induced *V*_o_. The stability of the oxygen vacancy tuned electrocatalysts was also monitored by a chronoamperometry test (Fig. 5f). Continuous running of the HER test for 50 h of Pl-30 (− 1.3 V vs SCE) was performed, and the overpotential variations at 10 mA cm^−2^ demonstrated the good durability of the catalyst, in which only a 2.3% increasing of the overpotential from 174 to 178 mV were recorded before and after the long-time durability test. Previous reports informed that it is difficult to maintain *V*_o_ vacancies promoted HER performance for long-term, as the *V*_o_ intends to be consumed during the HER process. Here, the plasma induced high *V*_o_ concentration was retained for achieving excellent catalysis stability. We also examined the morphology and surface composition variation of the catalysts after the stability test. All the microstructural and compositional characterizations, including XRD (Fig. S12), SEM (Fig. S13), and XPS (Fig. S14), indicated almost no changes occurred on Pl-30, except for partial reduction of Bi_2_O_3_ into metallic bismuth on the surface.

### DFT Calculations Study

**Fig. 6:**
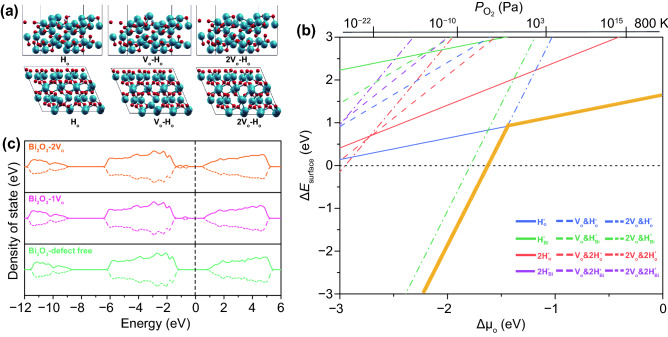
DFT calculations on the formation and stability of *V*_o_ within Bi_2_O_3_. From left to right, hydrogen atom adsorbed Bi_2_O_3_ (010) surface models in defect-free state and with one or two oxygen vacancy incorporated (**a**), the calculated relative surface energies as functions of the chemical potential of oxygen (∆*μ*_O_) (**b**), and the spin-polarized electronic density of states of Bi_2_O_3_ surfaces with different oxygen vacancies coverage (**c**)

Based on the above experimental results, it is interesting that a proper *V*_o_ modulation in the 2D Bi_2_O_3_ catalysts is very critical to performance. DFT calculations were, therefore, performed to understand the mechanism of the enhanced electrocatalytic HER performance in Bi_2_O_3_ with the variation of the oxygen vacancy concentration. The active hydrogen adsorption sites related with both Bi and O atoms were used to find the most stable adsorption sites by evaluating the surface formation free energies (Fig. 6).

The considered hydrogen intermediate adsorption types, as the representative models shown in Fig. 6a, include single H* on O site (H*_O_), single H* on single oxygen vacancy site (*V*_o_ − H*_Vo_), single H* on twin oxygen vacancies site (2*V*_o_ − H*_Vo_), single H* on Bi site (H*_Bi_), single H* on the bismuth site beside the single oxygen vacancy (*V*_o_ − H*_Bi_), single H* on the Bi site beside the twin oxygen vacancies site (2*V*_o_ − H*_Bi_), two H* on O site (2H*_O_), two H* on single oxygen vacancy site (*V*_o_ − 2H*_Vo_), two H* on twin oxygen vacancies site (2*V*_o_ − 2H*_Vo_), two H* on Bi site (2H*_Bi_), two H* on the bismuth site beside the single oxygen vacancy (*V*_o_ − 2H*_Bi_), and two H* on the Bi site beside the twin oxygen vacancies site (2*V*_o_ − 2H*_Bi_).

As shown in Fig. 6b, the calculated surface formation free energies of all types of possible hydrogen intermediate adsorption on the active sites of the Bi_2_O_3_ surface as the variation of the oxygen chemical potential (Δ*μ*_O_) or the oxygen partial pressure (*P*_O2_) were presented. In this plot, the oxygen chemical potential Δ*μ*_O_ (the bottom *x*-axis) is converted into the dependence on the oxygen partial pressure *P*_O2_ (the top *x*-axis) according to the ideal gas laws at a temperature of 800 K. Higher Δ*μ*_O_ and *P*_O2_ correspond to lower oxygen vacancy concentration in the catalysts. The energetically most stable hydrogen adsorbed models in the allowed chemical potential zone were also highlighted with thick yellow lines. In the high oxygen chemical potential range (Δ*μ*_O_ >  − 1.5 eV or *P*_O2_ > 10^2^ Pa), where the catalyst has very low concentration of oxygen vacancies, the hydrogen intermediate is preferred to attach onto the active O site rather than the Bi sites, and the most possible adsorption model is the single H* on O site (H*_O_). At a low oxygen chemical potential range (Δ*μ*_O_ < − 1.5 eV or *P*_O2_ < 10^2^ Pa), where a high oxygen vacancies concentration exists, the adsorption of single H* on twin oxygen vacancies site (2*V*_o_ − H*_Vo_) yields the lowest formation energy. While the high oxygen vacancy concentration leads to favorable and high number of H* adsorption, the stability of the 2*V*_o_ − H*_Vo_ significantly increases as the dramatic drop of the formation energy with the decrease of ∆*μ*_O_ It is confirmed that the *V*_o_ concentration cannot be too high, otherwise the desorption of H* becomes unfavorable and thus deactivate the HER catalysis. This result well explains the existence of the saturation of *V*_o_-induced activity in the 2D Bi_2_O_3_ nanosheets for triggering HER.

Figure 6c shows the calculated density of states (DOS) of Bi_2_O_3_ with different oxygen concentrations. A clear reduced band gap of the Bi_2_O_3_ resulted from the appearance of the defect states of oxygen vacancies at the top of valence band, which contributes to improved charge transfer required for the HER.

## Conclusion

In conclusion, 2D Bi_2_O_3_ nanosheets with modulated oxygen vacancies via the customized atmospheric-pressure plasma irradiation were studied for activated electrocatalytic HER in alkaline media, which cannot only balance the hydrogen intermediates adsorption but also improved the charge transfer kinetics. A linear relationship between the plasma-induced *V*_o_ concentration and the HER performance was revealed, ascribed by the optimized H* adsorption energy with improved charge transfer, before reaching a saturation concentration. After that, the increased *V*_o_ level resulted in trapped defects, which lowered the charge carrier density, and induced highly stable H* adsorption on the active sites, and thus decreased the catalytic efficiency. By tuning the oxygen vacancy concentration, the HER-inert Bi_2_O_3_ was activated into an active HER catalyst with a low overpotential of 174.2 mV to reach 10 mA cm^−2^, a low Tafel slope of 80 mV dec^−1^, a high exchange current density of 316 mA cm^−2^, and excellent stability. This work, thus, paves a way to activating inherently inert metal oxide materials into high performance electrocatalysts and provides some insights into the engineering of *V*_o_ for energy conversion and storage.

## Supplementary Information

Below is the link to the electronic supplementary material.

Supplementary file1 (PDF 949 KB)
