# Supplementary material for: Conversion of Catalytically Inert 2D Bismuth Oxide Nanosheets for Effective Electrochemical Hydrogen Evolution Reaction Catalysis via Oxygen Vacancy Concentration Modulation
Source: Nanomicro Lett. 2022 Apr 1;14:90. doi: 10.1007/s40820-022-00832-6 (PMC8975907; doi:10.1007/s40820-022-00832-6)
Supplement: Supplementary file 1 — Supplementary file1 (PDF 949 KB) [file 40820_2022_832_MOESM1_ESM.pdf]

Supporting Information for

Conversion of Catalytically Inert 2D Bismuth Oxide Nanosheets for Effective Electrochemical Hydrogen Evolution Reaction Catalysis via Oxygen Vacancy Concentration Modulation

Ziyang Wu[1], Ting Liao[1, 2, *], Sen Wang[3], Janith Adikaram Mudiyanselage[4], Aaron S. Micallef [4, 5], Wei Li[4], Anthony P. O'Mullane[2, 4], Jianping Yang[6], Wei Luo[6], Kostya (Ken) Ostrikov[2, 4], Yuantong Gu[1, 2] and Ziqi Sun[2, 4, *]

[1]School of Mechanical, Medical and Process Engineering, Queensland University of Technology, 2 George Street, Brisbane, QLD 4000, Australia

[2]Centre for Materials Science, Queensland University of Technology, 2 George Street, Brisbane, QLD 4000, Australia

[3]School of Earth and Atmospheric Sciences, Queensland University of Technology, 2 George Street, Brisbane, QLD 4000, Australia

[4]School of Chemistry and Physics, Queensland University of Technology, 2 George Street, Brisbane, QLD 4000, Australia

[5]Central Analytical Research Facility, Queensland University of Technology, 2 George Street, Brisbane, QLD 4000, Australia

[6]State Key Laboratory for Modification of Chemical Fibers and Polymer Materials, College of Materials Science and Engineering, Donghua University, Shanghai 201620, P. R. China

*Corresponding authors. E-mail: t3.liao@qut.edu.au (Ting Liao); ziqi.sun@qut.edu.au (Ziqi Sun)

Supplementary Figures and Tables

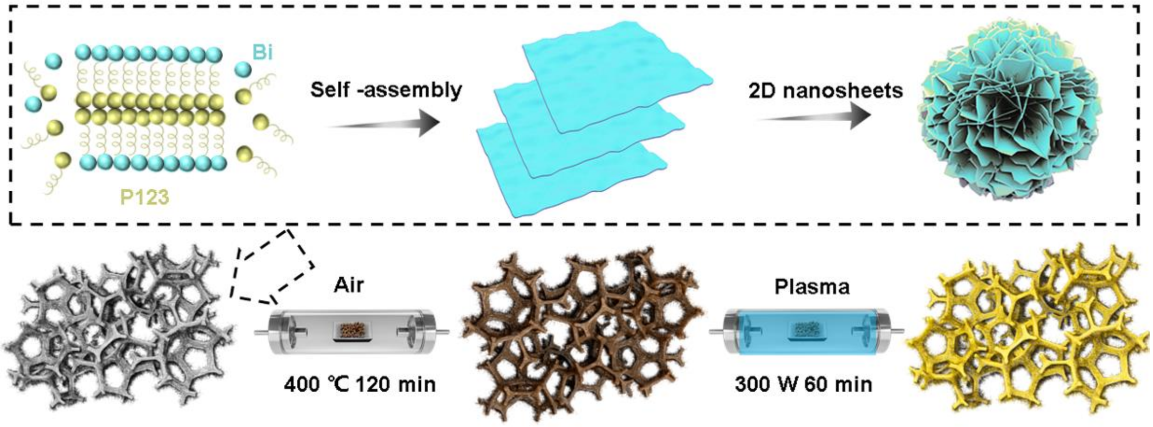

Fig. S1 The fabrication process of the N_2 plasma processed Bi_2O_3 sample on Ni foam

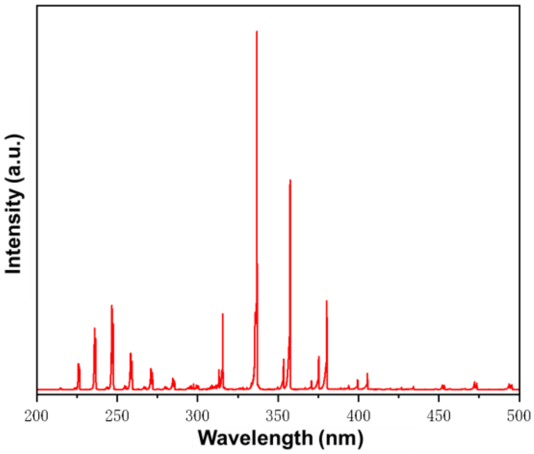

Fig. S2 OES spectra of the atmospheric pressure DBD plasma under nitrogen. Here, the bands corresponding to the second positive system of N_2 with the range of 300-400 nm [S1]

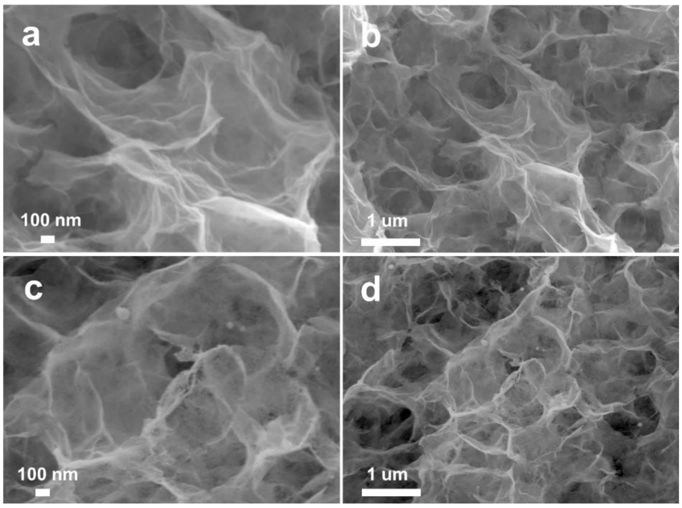

Fig. S3 SEM images of the sample after hydrothermal reaction (**a**, **b**) and the sample Pl-60 (**c**, **d**)

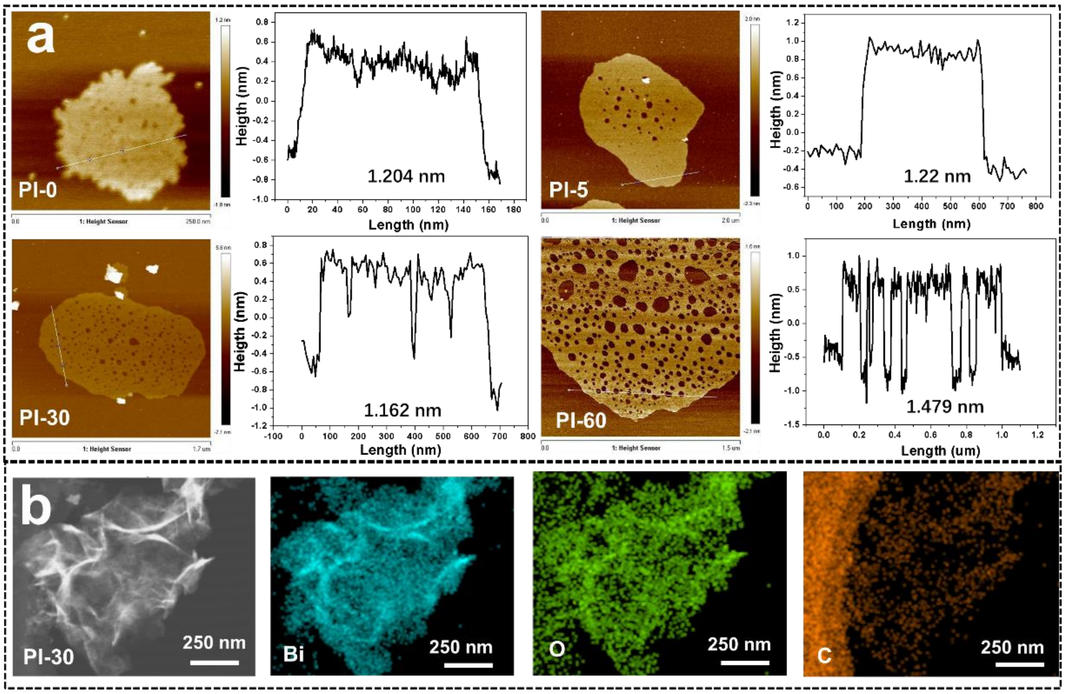

Fig. S4 AFM images of different samples and the EDS mapping image of sample Pl-30

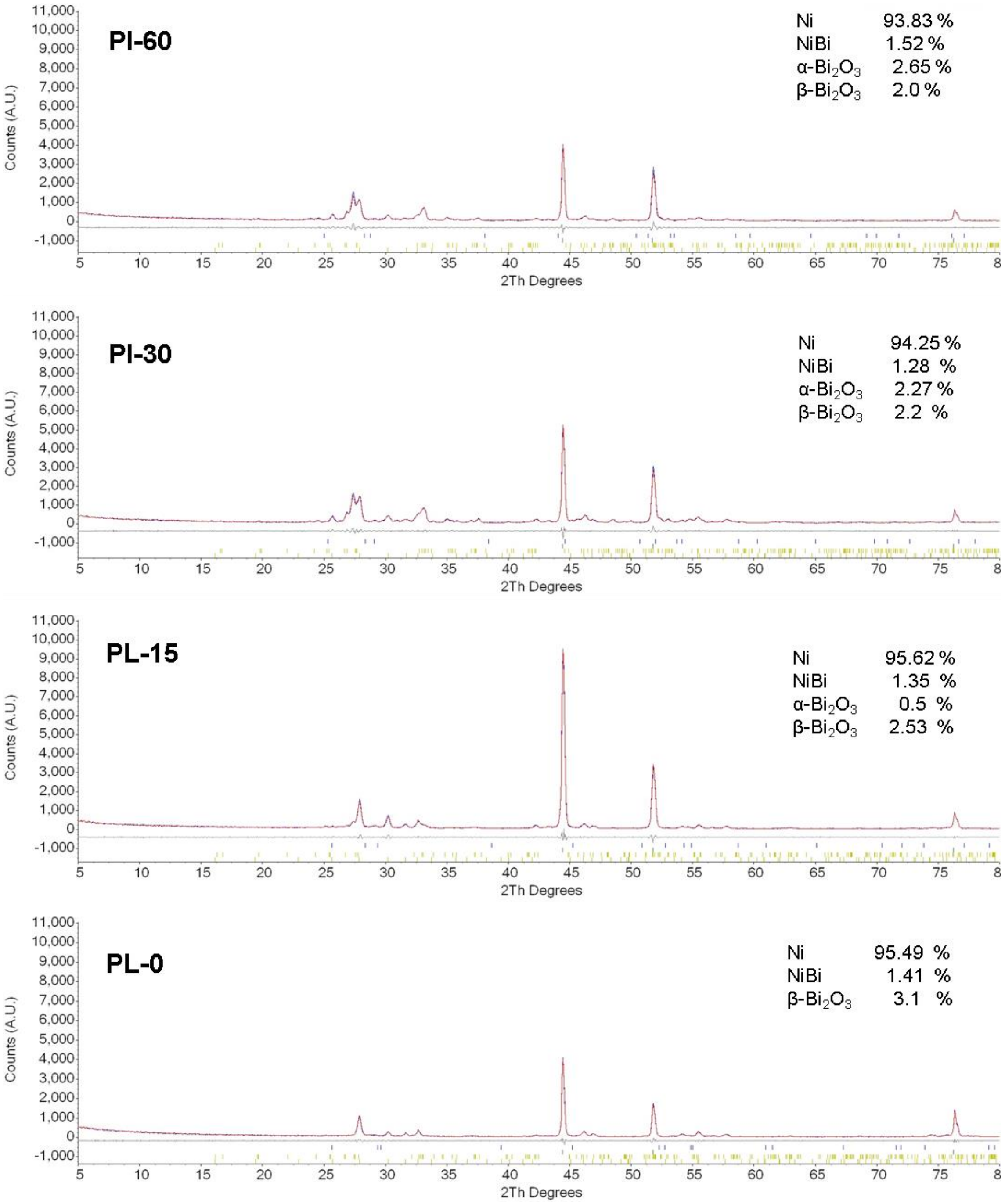

Fig. S5 The refined XRD surveys of the plasma processed samples

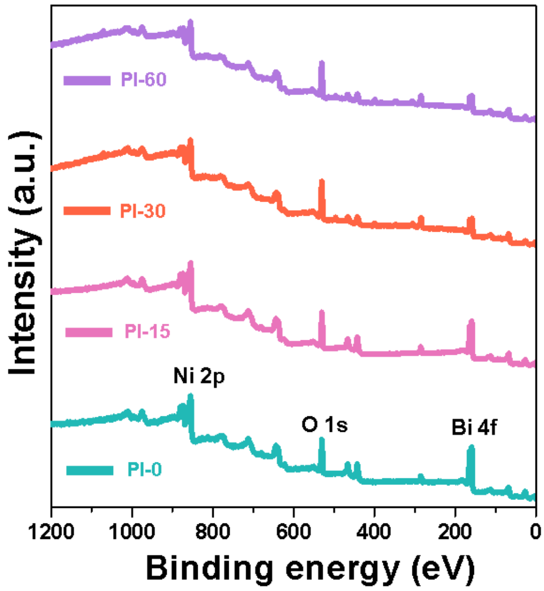

Fig. S6 Wide XPS spectrums of different samples

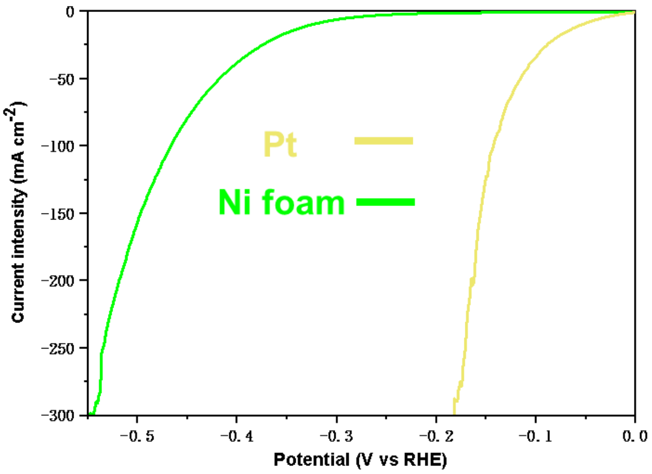

Fig. S7 The polarization curves of bare Ni foam and Pt/C loaded Ni foam with a scan rate of 5 mV s^{-1}

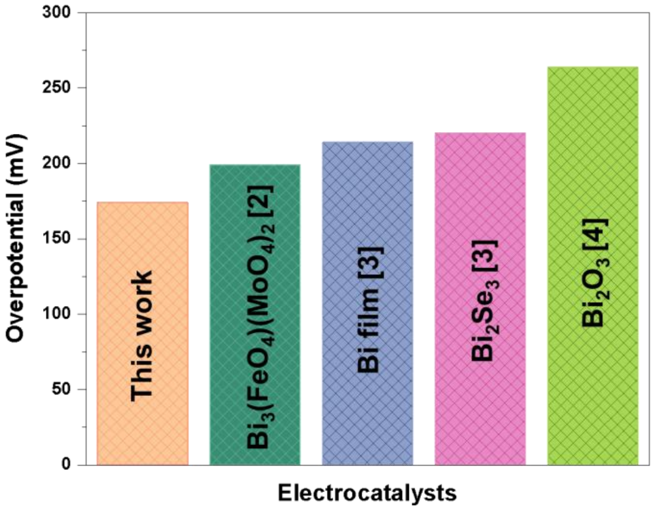

Fig. S8 The comparation of HER performance of bismuth based electrocatalysts [S2-S4]. As very few Bismuth-based electrocatalysts was employed for alkaline HER, only three references were listed here

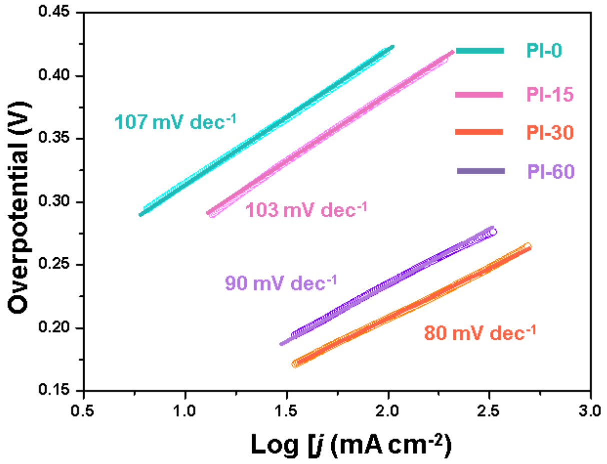

Fig. S9 The simulated Tafel slopes for different samples

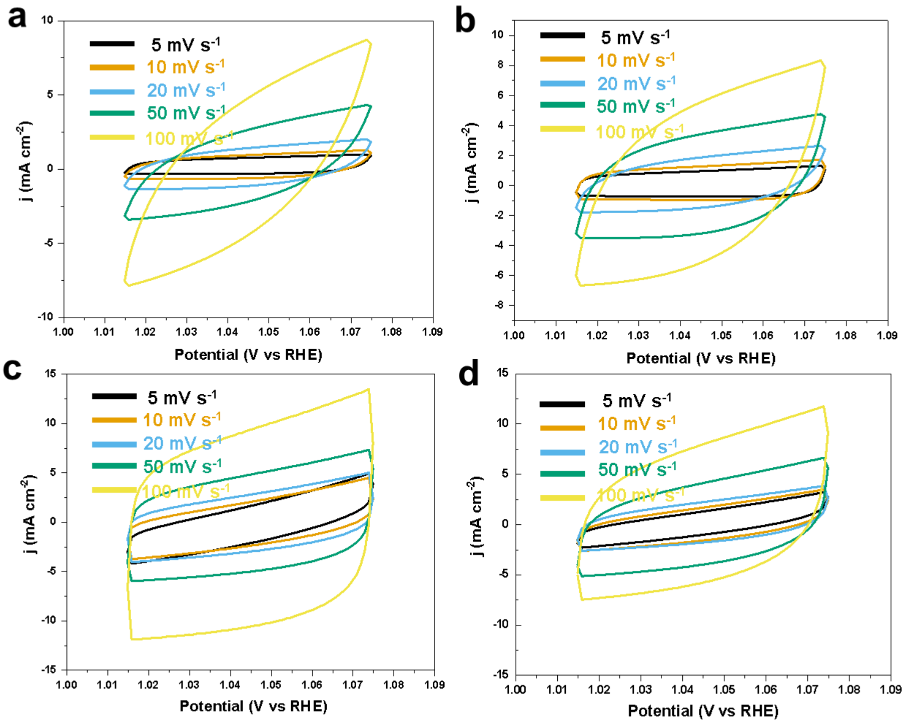

Fig. S10 The cyclic voltammetry (CV) cycles in the region between 1.01 and 1.08 V (vs. Ag/AgCl) at different scan rates (5, 10, 20, 50, and 100 mv s^{-1}) of Pl-0 (**a**), Pl-15 (**b**), Pl-30 (**c**) and Pl-60 (**d**)

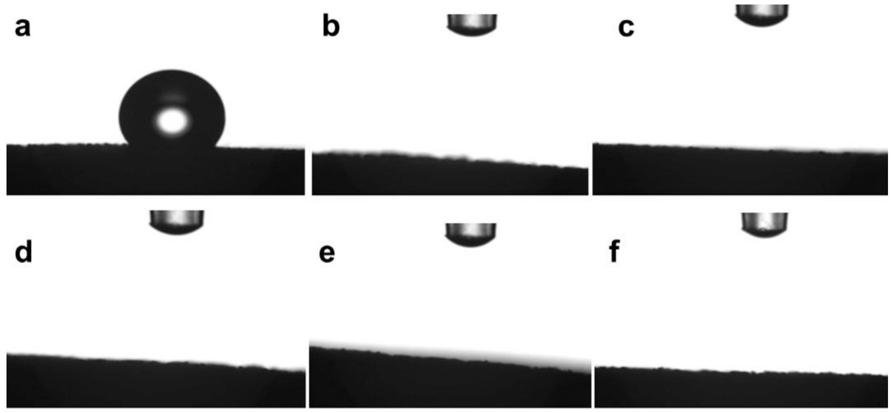

Fig. S11 The wetting ability Ni foam (**a**), the sample after hydrothermal reaction (**b**), Pl-0 (**c**), Pl-15 (**d**), Pl-30 (**e**) and Pl-60 (**f**)

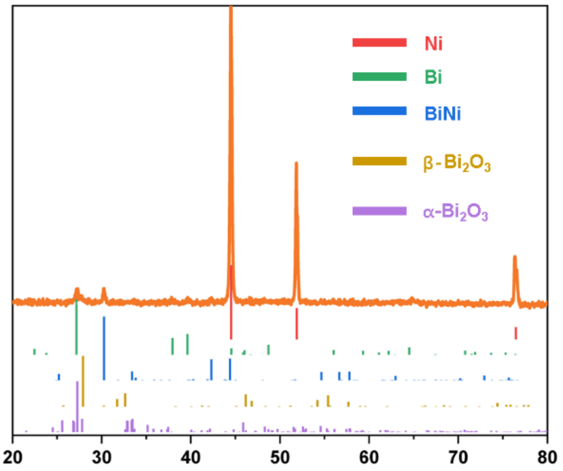

Fig. S12 XRD spectrum of sample Pl-30 after HER durability test. The corresponding PDF cards are Ni-PDF#04-004-6807, Bi-PDF#04-007-9968, BiNi-PDF#04-003-5243, α-Bi_2O_3-PDF#04-017-2112 and β-Bi_2O_3-PDF#04-008-7003

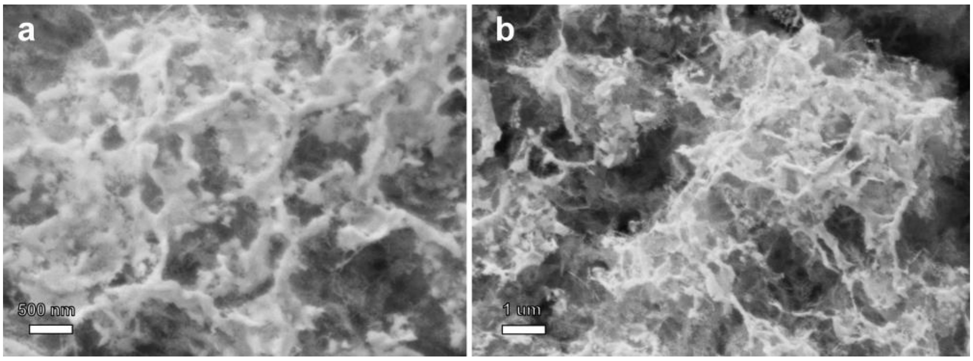

Fig. S13 SEM images of sample Pl-30 after HER durability test

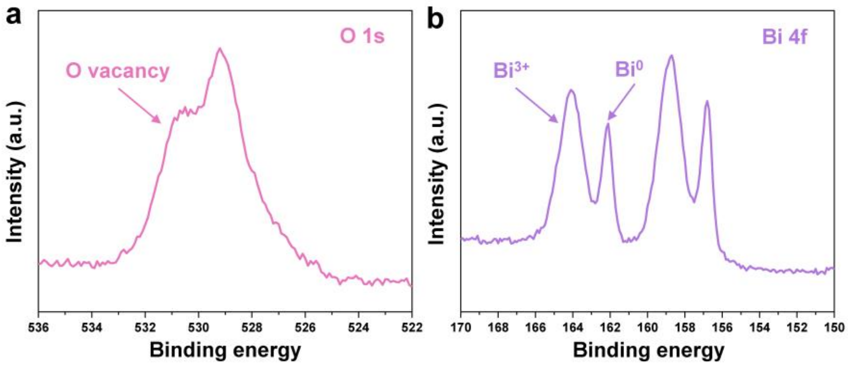

Fig. S14 XPS of Pl-30 after HER durability test

Table S1 The contents of V_o calculated from the XPS data

XPS	Pl-0	Pl-15	Pl-30	Pl-60
**Value (%)**	19.7	26.5	44.1	49.0
**Increase (%)**	0	6.8	24.4	29.3
**Ratios (/Pl-0)**	1	1.34	2.24	2.49

Table S2 The intensity of V_o signal calculated from the EPR data

EPR	Pl-0	Pl-15	Pl-30	Pl-60
**Value (a.u.)**	50	977	3050	3885
**Ratios (/Pl-0)**	1	19.5	61	77.7

Table S3 The contents of carrier density derived from the M-S data

M-S	PI-0	PI-15	PI-30	PI-60
**Slope**	32.2	21.9	5.37	6.09
**Value (cm^{-3})**	2.53×10^{23}	3.71×10^{23}	1.52×10^{24}	1.34×10^{24}
**Ratios (/PI-0)**	1	1.47	6.0	5.29

Supplementary References

[S1] J. Weerasinghe, S. Sen, J.M.K.W. Kumari, M.A.K.L. Dissanayake, G.K.R. Senadeera et al., Efficiency enhancement of low-cost metal free dye sensitized solar cells via non-thermal atmospheric pressure plasma surface treatment. Solar Energy **215**, 367-374 (2021). https://doi.org/10.1016/j.solener.2020.12.044

[S2] S. Khatun, P. Roy, Bismuth iron molybdenum oxide solid solution: a novel and durable electrocatalyst for overall water splitting. Chem. Commun. **56**(53), 7293-7296 (2020). https://doi.org/10.1039/D0CC01931C

[S3] S. Razzaque, M.D. Khan, M. Aamir, M. Sohail, S. Bhoyate et al., Selective synthesis of bismuth or bismuth selenide nanosheets from a metal organic precursor: investigation of their catalytic performance for water splitting. Inorg. Chem. **60**(3), 1449-1461 (2021). https://doi.org/10.1021/acs.inorgchem.0c02668

[S4] Z. Wu, J. Mei, Q. Liu, S. Wang, W. Li et al., Phase engineering of dual active 2D Bi_2O_3-based nanocatalysts for alkaline hydrogen evolution reaction electrocatalysis. J. Mater. Chem. A **10**(2), 808-817 (2021). https://doi.org/10.1039/D1TA09019D
